# Two female siblings with West syndrome: Familial idiopathic West syndrome with genetic susceptibility and variable phenotypic expression

**DOI:** 10.4103/1817-1745.76116

**Published:** 2010

**Authors:** Ahmet Okay Caglayan, Hakan Gumus, Mitsuhiro Kato

**Affiliations:** Department of Medical Genetics, Kayseri Education and Research Hospital, Kayseri, Turkey; 1 Department of Pediatrics, Erciyes University, Kayseri, Turkey; 2 Department of Pediatrics, Medical Faculty, Yamagata University, Yamagata, Japan

**Keywords:** Genetics, West syndrome, *CDKL5* gene, new genes

## Abstract

The West syndrome (WS) is a characteristic form of epilepsy which usually begins in the first year of life. We describe two female siblings, aged 4 and 2 years, respectively, born from third degree consanguineous parents, with infantile spasms and developmental delay. Epileptic spasms had not a good outcome under antiepileptic drug treatment. Clinical and imaging features were of different severity in both siblings. Routine biochemical tests, metabolic investigations, and chromosomal analysis were normal. We analyzed *CDKL5* gene by direct sequences and denaturing high-performance liquid chromatography using Transgenomic WAVE system. Analysis of the *CDKL5* gene, which is responsible for female patient with WS, did not show any disease-causing mutation. WS has heterogeneous backgrounds, and may be more than one gene is responsible for its familial forms. In this family, consanguinity is observed in parents, which usually suggests that autosomal recessive inheritance is likely.

## Introduction

The association of infantile spasms, an electroencephalogram (EEG) abnormality often called hypsarrhythmia, and psychomotor deterioration is called West syndrome (WS) that begins in the first year of life, most commonly between 4 and 8 months of age. It occurs in 0.16 to 0.42 per 1000 live births and is divided into ‘cryptogenic’ (if no previous illness is apparent), or ‘symptomatic’ (if pre-existing disorders affecting the brain are present) by ILAE commission report.[1]

Approximately 50% of WS cases are associated with a number of disorders such as, cerebral palsy, Down’s syndrome, tuberous sclerosis, neurofibromatosis, incontinentia pigmenti, neuronal migration disorders, etc. However, in a significant minority of cases, the etiology remains unknown.[2] Approximately two-thirds of affected infants will have a detectable underlying neurological abnormality, but still little is known about the pathophysiological basis for infantile spasms, and treatment remains problematic. The syndrome is usually sporadic and familial cases have been infrequent. Vigevano *et al*. reported that among the children classified as having a cryptogenic WS, many–in their series at least 55%–fulfill the criteria for an idiopathic etiology.[3] Girls with unexplained WS should be examined for a mutation in the *CDKL5* gene.[2] In this study, we described two female patients with WS having no mutation in the *CDKL5* gene.

## Case Report

### Case 1

The index patient was the first child of third degree consanguineous parents, born at term, after an uneventful pregnancy and delivery (birth weight, 2980 g, 25-50^th^ percentile; length, 50 cm, 75^th^ percentile; head circumference, 37 cm, 50^th^ percentile). Family history for epilepsy and/or mental retardation was unremarkable. At the age of 5 months, she developed infantile spasms and a hypsarrhythmic EEG pattern. She responded to neither vigabatrin nor ACTH. At the age of 4 years, she had microcephaly and mental retardation. She was neither able to sit without support nor grasp objects. She had no expressive speech and had no eye contact. Her metabolic tests, routine biochemical tests, electric response audiometry (ERA) test, and chromosome analysis were normal. Visual evoked response (VER) test and fundoscopy showed optical atrophy in the left eye. Neuroimaging (magnetic resonance imaging [MRI]) revealed global brain atrophy.

### Case 2

The younger sister of the index patient was born at term, after an uneventful pregnancy and delivery (birth weight, 3300 g, 50-75^th^ percentile; length, 52 cm, 50-75^th^ percentile; head circumference, 36 cm, 50^th^ percentile). At the age of 4 months, she developed infantile spasms and hypsarrhythmic EEG pattern like her elder sister. She did not respond to vigabatrin and ACTH. At the age of 2 years, she was able to sit but could not walk, and was mentally retarded. She had eye contact but no spoken word, grasping objects was very difficult. Her metabolic tests, routine biochemical tests, ERA test, chromosome analysis, VER test, and neuroimaging (MRI) were normal.

### Molecular studies

We have done a mutation screening test for the *CDKL5* gene with an informed consent from their parents. Briefly, methods are as follows. Genomic DNA was extracted according to standard procedures and amplified for all coding exons (exon 1-21) and flanking introns of *CDKL5*. The primers and PCR conditions are available on request. Direct sequence analysis for the PCR product of exon 4 was performed on an automated DNA sequencer (ABI 310; Applied Biosystems, Foster City, CA). Other products were analyzed by denaturing high-performance liquid chromatography using the Transgenomic WAVE system (Transgenomic, Omaha, NE). Both the children had not carried any disease-causing mutation in the *CDKL5* gene.

## Discussion

Previously, Reiter *et al*. reported two families, each with occurrence of WS in two siblings, and they suggested that these cases may be classified as ‘familial idiopathic West syndrome‘ genetic susceptibility and variable phenotypic expression, such as our cases.[4] Recently, two novel genes, *ARX *(*Aristaless*-related homeobox) and *CDKL5/STK9* (Cyclin-dependent kinase-like 5), have been found to be responsible for cryptogenic WS or infantile spasms (also known as X-linked infantile spasm syndrome-1 [ISSX1] [MIM:308350] and -2 [ISSX2] [MIM:300672], respectively). Both are located in the human chromosome Xp22 region and are mainly expressed and play roles in fetal brain. Boys with unexplained WS should be examined for a mutation in the *ARX* gene, especially, when the family history is positive for mental retardation and epilepsy. Girls with unexplained WS should be examined for a mutation in the *CDKL5* gene, which is inherited X-linked dominant, especially, when the family history is positive for mental retardation and epilepsy and clinical findings consist of severe phenotype of early-onset infantile spasms, global developmental arrest, and profound mental retardation.[2,5] In addition to these genes, rarely other disease-causing genes are described, such as the *SLC25A22* gene mutations responsible for infantile spasm syndrome-3 (MIM:609304) and the *STXBP1* gene mutations in patients with neonatal or infantile onset of tonic-clonic or tonic seizures, suppression-burst pattern on EEG, profound mental retardation, and MRI evidence of hypomyelination, responsible for the infantile spasm syndrome-4 (MIM:612164).[6,7]

Lastly, Sugai *et al*. reported result of a multicenter study with ‘Nationwide survey on familial cases of WS in first- and second-degree relatives‘ from Japan and they reviewed the literature.[8] According to their study, familial cases of WS have characteristic clinical features and genetic mechanisms. Long-term seizure and developmental prognoses were far better than those in WS in general, with seizure-free rate of 82% and normal mental development rate of 44%. Poor prognosis was detected, limited to specific symptomatic cases; the familial cases well responded to antiepileptic drugs. Previously, among patients with proximal interstitial deletions of 7q, the findings of abnormal EEG and/or seizures are reported to be associated with deletion of the region 7q21.[9] Recently, Marshall *et al*. identified a locus for WS by high-resolution mapping of 7q11.23-q21.1 interstitial deletions in patients. These children exhibit very delayed motor and developmental milestones compared to children with typical Williams-Beuren syndrome, often in combination with hypotonia and severe intellectual disability.[10] However, our patient‘s clinical findings do not resemble of Williams-Beuren syndrome.

It is well known that the outcome of WS depends mainly on etiology.[3,11,12] In this family, consanguinity is observed in parents, which usually suggests that autosomal recessive inheritance is likely. Although generally significantly better outcome, in terms of both mental development and seizure remission, was observed in children in the cryptogenic and idiopathic groups than in the symptomatic group, prognosis of our case was not good and further large number case studies should be examined for detecting specific genetic background in these patient‘s group.[12,13]
